# “Moderate” adjuvant chemotherapy-induced leukopenia is beneficial for survival of patients with early breast cancer: a retrospective study

**DOI:** 10.1186/s12885-023-11680-x

**Published:** 2023-12-13

**Authors:** Li Wang, Chang Jiang, Na Wang, Yan-Ling Wen, Si-Fen Wang, Cong Xue, Xi-Wen Bi, Zhong-Yu Yuan

**Affiliations:** Department of Medical Oncology, State Key Laboratory of Oncology in South China, Collaborative Innovation Center for Cancer Medicine, Guangdong Provincial Clinical Research Center for Cancer, Sun Yat-sen University Cancer Center, 651 Dongfeng Road East, Guangzhou, 510060 People’s Republic of China

**Keywords:** Survival, Nomogram, Early Breast cancer, Chemotherapy-induced leukopenia

## Abstract

**Background:**

The association between chemotherapy-induced leukopenia (CIL) and survival for patients with early breast cancer (EBC) is not known. We investigated the relationship between different grades of CIL and survival in patients with EBC receiving adjuvant chemotherapy.

**Methods:**

A total of 442 patients with EBC receiving a regimen containing an anthracycline (A) and taxane (T) were included into our analysis. Survival analyses were undertaken using Kaplan–Meier curves. The P-value was calculated using the log rank test. Subgroup analysis was conducted to investigate the correlation of CIL grade and survival based on the clinicopathological characteristics of patients. Afterwards, univariate and multivariate analyses screened out independent prognostic factors to construct a prognostic model, the robustness of which was verified.

**Results:**

Patients with EBC who experienced grade 2–4 (“moderate” and “severe”) CIL were associated with longer overall survival (OS) than those with grade 0–1 (mild) CIL (*P* = 0.021). Compared with patients with mild CIL, OS was longer in patients with severe CIL (*P* = 0.029). Patients who suffered from moderate CIL tended to have longer OS than those with mild CIL (*P* = 0.082). Nevertheless, there was no distinguishable difference in OS between moderate- or severe-CIL groups. Subgroup analysis revealed that patients with moderate CIL had longer OS than those with mild CIL among patients who were premenstrual, or with human epidermal growth factor receptor 2-positive (HER2+), > 3 lymph nodes with metastases, a tumor diameter > 5 cm. A prognostic model based on menstrual status, N stage, and CIL grade showed satisfactory robustness.

**Conclusion:**

The grade of CIL was strongly associated with the prognosis among patients with EBC who received a regimen containing both anthracyclines and taxanes. Patients with a “moderate” CIL grade tended to have better survival outcomes.

**Supplementary Information:**

The online version contains supplementary material available at 10.1186/s12885-023-11680-x.

## Background

 Breast cancer (BC) has surpassed lung cancer as the most prevalent cancer type among women in 185 countries, with approximately 65% of BC cases diagnosed in the early stages (I–III) [1]. Despite the 5-year survival rate of BC patients exceeding 85%, nearly 30% of patients with early breast cancer (EBC) will experience local recurrence and/or distant metastasis, resulting in physical and psychological suffering and an unfavorable prognosis [2, 3].

Several studies have reported the effectiveness of adjuvant chemotherapy in relieving pain, delaying potential relapse and/or metastasis, and significantly improving survival among patients with early-stage breast cancer (EBC) [4, 5]. EBC patients have a variety of chemotherapy options and regimens available, including anthracycline (A) and taxane (T), which are commonly used in clinical practice [6–8]. The specific dosages of A and T are typically calculated based on the patient’s body surface area (BSA), with adverse effects (AEs) occurring frequently in individuals undergoing adjuvant chemotherapy, resulting in discomfort and reluctance to continue treatment [9]. Therefore, in pursuit of more individualized and precise therapy, it is necessary to determine suitable therapeutic doses for each EBC patient to improve survival rates and minimize potential toxicity.

Regimens including A and T often result in many adverse events (AEs), including myelosuppression, alopecia, nausea, vomiting, peripheral neurotoxicity, and hand and foot syndrome [10–13]. The occurrence rate of myelosuppression is particularly high in these patients because it results in a decrease in bone marrow activity which then leads to a decline in red blood cells (RBC), white blood cells (WBC), and platelets (PLT). Furthermore, severe myelosuppression increases the incidence of febrile neutropenia and poses a significant threat to patients’ survival [14–16]. To effectively reduce harmful effects of myelosuppression, symptomatic treatments (e.g., injection of granulocyte colony-stimulating factor (G-CSF)) and dose reduction are employed [17, 18]. However, the administration of G-CSF has been reported to promote cancer progress, and excessive dose reduction is also not beneficial to control tumor progress as treatment efficacy can only be guaranteed if the drug dose is above 85% of the standard dose [19, 20].

The total lifespan of leukocytes is typically 13 to 20 days, so chemotherapy-induced leukopenia (CIL) can be detected readily and early in blood routine examination. Therefore, it may be feasible to explore the potential association between CIL and the effectiveness of adjuvant chemotherapy and adjust drug dosages accordingly. RECOURSE and J003 trials of metastatic colorectal cancer shown that trifluridine/tipiracil-treated patients who developed chemotherapy-induced neutropenia (CIN) had significantly better survival rates than those who did not develop CIN [21, 22]. However, a previous study concluded that CIN was not a significant prognostic indicator for ovarian cancer patients treated with paclitaxel/carboplatin as first-line chemotherapy [23, 24]. Hence, further investigations are required to investigate the relationship between CIL and patients’ survival. In this study, we aim to explore the association between CIL and survival in patients with EBC who have been treated using a combination of A and T regimen.

## Methods

### Enrollment

This retrospective study involved a cohort of 442 patients who were first diagnosed with EBC and received combined regimens of A and T at Sun-Yat Sen University Cancer Center (SYSUCC; Guangzhou, China). The time interval of the initial diagnosis was from 1 January 2012 to 31 December 2017. Only patients who underwent all cycles of chemotherapy in SYSUCC were eligible for inclusion. The detailed information on inclusion and exclusion criteria are presented in Supplementary Appendix 1.

After a thorough screening process, 442 patients were found to be eligible for study inclusion and underwent retrospective assessment. These patients were subsequently categorized into various subgroups based on their clinicopathological characteristics.

### Data collection and grouping of patients

We searched the electronic medical records system of SYSUCC to retrieve the clinicopathological characteristics of patients. Information for the age at the diagnosis, height, weight, lymphatic-vessel invasion, T stage, N stage, receptor status (estrogen receptor (ER), progesterone receptor (PR), human epidermal growth factor receptor 2 (HER2)), Ki67 score (stained with MIB1-mindbomb E3 ubiquitin protein ligase 1, monoclonal antibody and assessed by two independent pathologists), and pathological grade (PG) was extracted. All patients were comprehensively examined (history-taking, physical examination, general laboratory tests, imaging) before treatment.

In this retrospective study, ER- or PR-positive patients with BC were those with > 1% expression of ER or PR, respectively, in nuclei according to immunohistochemistry (IHC) [25]. With regard to HER2 status, tumor cells scoring 3 + or 2 + upon IHC but with an amplified Erb-B2 receptor tyrosine kinase 2 (ERBB2) gene detected by fluorescence in situ hybridization (FISH) were defined as being HER2-positive [26]. Blood counts were documented routinely in each chemotherapy cycle. The most severe leukopenia during chemotherapy was included in the analysis to avoid the deviation caused by different detection values. According to Common Terminology Criteria for Adverse Events v5.0, the degree of CIL was categorized into five grades (0, 1, 2, 3, 4) and recorded in figures and tables as 0, I, II, III, and IV, respectively. Patients who experienced grade-1 CIL often do not require specific clinical intervention, so we integrated patients without CIL and those with grade-1 CIL into “mild CIL” (grade 0–1). Prophylactic G-CSF or drug reduction was given to patients with grade-4 CIL, whereas these interventions were not necessary for patients with grade-2–3 CIL. Taking these factors into consideration, we classified CIL into three groups: “mild” (grade 0–1), “moderate” (grade 2–3), and “severe” (grade 4).

### Primary endpoints and follow-up

Overall survival (OS) refers to the duration from the diagnosis to death from any cause or to the final follow-up. Relapse-free survival (RFS) denotes the period from surgery until the recurrence of local or regional draining lymph nodes, or local and regional secondary syngeneic neoplastic lesions. Patients were tracked through regular outpatient appointments or telephone interviews.

### Statistical analyses

A continuous variable, age, was transformed into a categorical variable using the median value as the cutoff. Categorical variables were reported in terms of frequencies and percentages. The rank sum test was employed to investigate the correlation between the CIL grade and other clinicopathological variables.

Cox proportional hazards matrices were utilized for both univariate and multivariate analyses. Variables with *P* < 0.2 (two-tailed) from the univariate Cox regression analysis were included in the proportional hazards (PH) test. In the PH test, variables with P > 0.05 were considered to have no effect on survival over time, and were deemed acceptable for the COX proportional hazards model. *P* < 0.05 (two-tailed) was the threshold for statistical significance in multivariate analyses. Using R 4.1-0’s “rms” package (R Institute for Statistical Computing, Vienna, Austria), we developed a prognostic nomogram for 5- and 8-year overall survival by combining results from univariate and multivariate analyses. Then, the robustness of the predictive model was tested from two dimensions of discrimination (concordance index (C-index) and receiver operating characteristic (ROC) curves) and accuracy (calibration curves). Finally, decision curve analysis (DCA) was conducted to evaluate the net benefit at different threshold probabilities and determine the clinical value of the model.

To evaluate the survival of patients stratified by different risk categories according to the predictive model, the following formula was used to count the risk score: risk score = e ^sum (every prognostic factor×corresponding coefficient)^. The median risk score was used to categorize patients into either high- or low-risk group. Subsequent analyses of overall survival were performed respectively for the low-risk and high-risk groups.

## Results

### Characteristics of patients

A total of 442 patients diagnosed with EBC were finally included in this study. The association between the grade of CIL and other clinicopathological features of all patients was presented in Table 1. The median age of patients in the study cohort was 46 (range: 28–71) years. The body mass index (BMI) revealed that 21.72% of patients were overweight (BMI > 25 kg/m^2^). Additionally, almost two-thirds of patients were premenopausal and over half tested positive for hormone receptors. Additionally, nearly two-thirds of the patients had a high Ki-67 index. According to the tumor–node–metastasis (TNM) system, 59.05% patients were in T2 stage. The number of patients in stage N0 (150) was comparable to that in stage N1 (141). Furthermore, the majority (71.04%) of patients received the AC-T regimen for adjuvant chemotherapy.

**Table 1:** The relationship between CIL grade and other clinicopathological features

Baseline Characteristics	Total(n,%)	CIL Grade(n,%)			*P*-value
		0/I	II/III	IV	
**Age at diagnosis**					0.215
≤ 46 years	224(50.68)	20(43.48)	145(55.77)	59(43.38)	
> 46 years	218(49.32)	26(56.52)	115(44.23)	77(56.62)	
**BMI**					0.860
< 25	346(78.28)	33(71.73)	208(80.00)	105(77.21)	
≥ 25	96(21.72)	13(28.26)	52(20.00)	31(22.79)	
**Menstrual Status**					0.185
premenopausal	280(63.35)	31(67.39)	169(65.00)	80(58.82)	
postmenopausal	162(36.65)	15(32.61)	91(35.00)	56(41.18)	
**HR Status**					0.668
Negative	162(36.65)	10(21.74)	105(40.38)	47(34.56)	
Positive	280(63.35)	36(78.26)	155(59.62)	89(65.44)	
**HER2 Status**					0.181
Negative	260(58.82)	32(69.57)	152(58.46)	76(55.88)	
Positive	182(41.18)	14(30.43)	108(41.54)	60(44.12)	
**KI67 Index**^a^					0.913
≤ 15%	103(23.30)	13(28.26)	56(21.54)	34(25.00)	
> 15%	339(76.70)	33(71.73)	204(78.46)	102(75.00)	
**Pathological Grade**					0.982
1 + 2	220(49.77)	27(58.69)	122(46.92)	71(52.21)	
3	222(50.23)	19(41.30)	138(53.08)	65(47.79)	
**T Stage**^b^					< 0.01
T1	152(34.39)	12(26.09)	83(31.92)	57(41.91)	
T2	261(59.05)	30(65.22)	157(60.38)	74(54.41)	
T3	29(6.56)	4(8.70)	20(7.69)	5(3.68)	
**N Stage**^b^					< 0.01
N0	150(33.94)	16(34.78)	87(33.46)	47(34.56)	
N1	141(31.90)	12(26.09)	90(34.62)	39(28.68)	
N2	81(18.33)	11(23.91)	39(15.00)	31(22.79)	
N3	70(15.83)	7(15.22)	44(16.92)	19(13.97)	
**LVI**					0.540
No	225(50.90)	21(45.65)	133(51.15)	71(52.21)	
Yes	217(49.10)	25(54.35)	127(48.85)	65(47.79)	
**Chemotherapy**					< 0.01
TE or TEC	69(15.61)	17(36.96)	24(9.23)	28(20.59)	
FEC-T	59(13.35)	8(17.39)	33(12.69)	18(13.24)	
AC-T	314(71.04)	21(45.65)	203(78.08)	90(66.18)	

*Abbreviations*: *CIL *Chemotherapy-induced leukopenia, *BMI *Body mass index, *HR *Hormone receptor, *HER-2 *Human epidermal growth factor receptor-2, *LVI *Lymphatic vessel invaded, *TE *Taxanes combined with Anthracycline, *TEC *Taxanes, Anthracycline combined with cyclophosphamide, *FEC-T *5-fluorouracil, Anthracycline combined with cyclophosphamide followed by Taxanes, *AC-T *Taxanes combined with cyclophosphamide followed by Taxanes

^a^Indicating DNA synthetic activity as measured using immunocytochemistry

^b^According to the 7th edition of the UICC/AJCC staging system

### CIL grade was closely correlated with OS

**Fig. 1:**
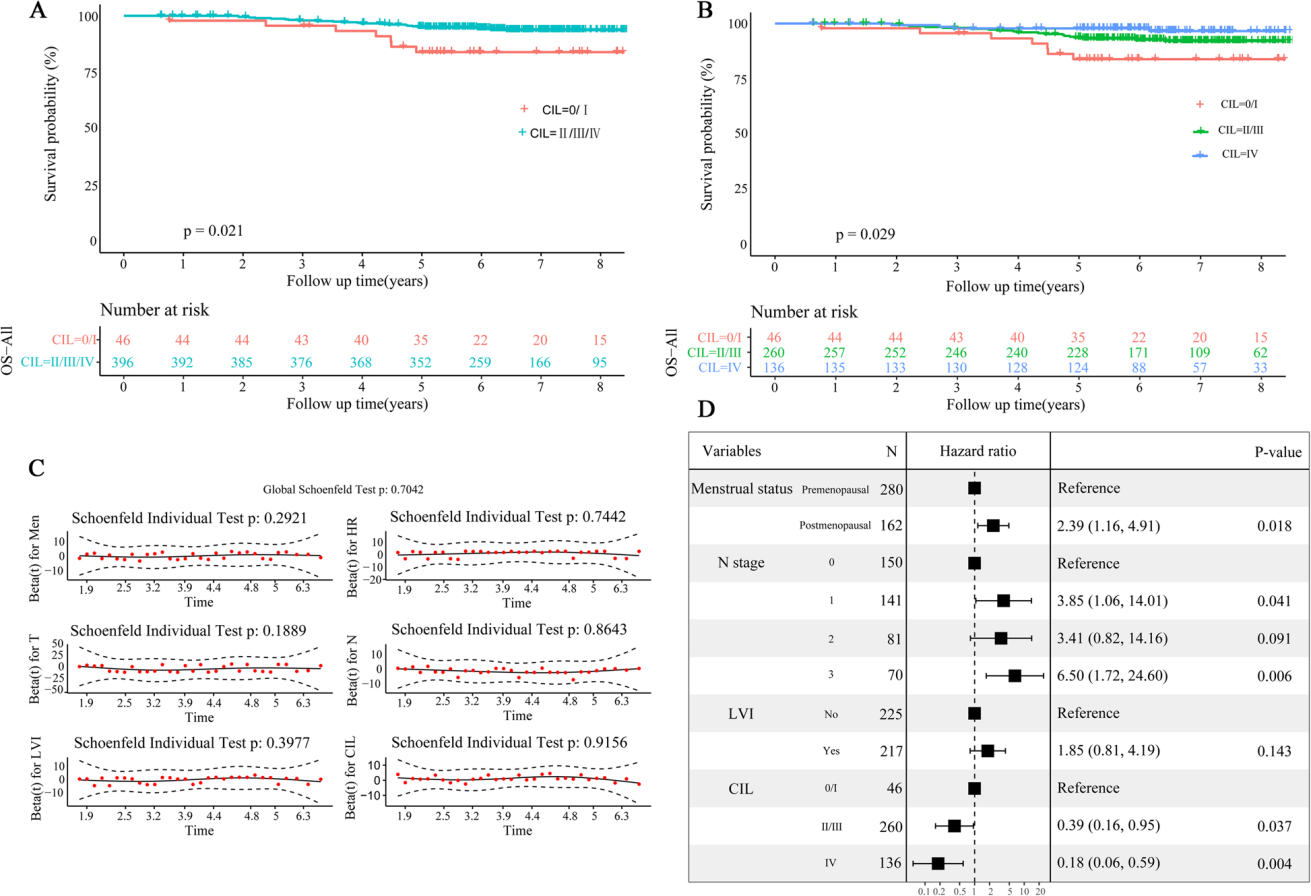
Development of the prognostic signature. **A** Kaplan-Meier curves for the overall survival (OS) of patients based on chemotherapy-induced leukopenia (CIL) grouping with grade 1 of CIL as the cut-off value. **B** Kaplan-Meier curves for the OSof patients based on chemotherapy-induced leukopenia (CIL) grouping as “mild” (grade 0–1), “moderate” (grade 2–3), and “severe” (grade 4). **C** The proportional hazards (PH) test of cohort dataset for the OS. **D** Results of the stepwise multivariate Cox regression analysis for the OS.

The CIL grade demonstrated a robust association with the OS of patients with EBC who underwent treatment with A and T. Patients experienced moderate to severe CIL (grade 2–4) showed significantly better OS than those with mild (grade 0–1) CIL (*P* = 0.021) (Fig. 1A). The estimated 5-year OS rates for grade 2–4 and grade 0–1 CIL were 94.8% (95% CI: 92.5-97%) and 83.9% ( 95% CI: 73.6-95.6%), respectively. The group with a CIL of moderate to severe degrees (grade 2–4) was further divided into more specific subgroups, revealing that the OS rate of the severe CIL was superior to that of the mild CIL (*P* = 0.029) (Fig. 1B). A tendency was observed that the OS rate of the moderate CIL was also better than mild CIL (*P* = 0.082). However, there was no significant statistical difference in OS between the moderate and the severe CIL groups (*P* = 0.164). Interestingly, patients who experienced mild, moderate, or severe CIL had similar RFS (mild vs. moderate: *P* = 0.862; mild vs. severe: *P* = 0.577; moderate vs. severe: *P* = 0.287).

### Relationship between CIL and survival in subgroup analyses

Table 2 shows the relationship between CIL grade and OS. Compared with the mild-CIL group, patients characterized as premenstrual, HER2+, in N2/N3 stage or T3 stage in moderate- and severe-CIL groups had longer OS. The difference between moderate- and severe-CIL groups for these parameters was not significant, which was in line with the survival analysis based on all participants. Hormone receptor-positive patients moderate CIL had shorter OS than severe CIL counterparts (HR = 4.439, 95%CI = 1.015–19.413, *P* = 0.048), while there was no obvious difference between moderate- and mild-CIL groups (HR = 0.466, 95%CI = 0.189–1.149, *P* = 0.097). Table 3 reflected that there was no significant association between CIL grades and RFS.

**Table 2:** The relationship between the grade of CIL and overall survival (OS)

Characteristics	Number(n,%)	Events(n,%)	II/III vs. 0/I		II/III vs. IV		IV vs. 0/I	
			HR, 95%CI	*P*-value	HR, 95%CI	*P*-value	HR, 95%CI	*P*-value
**Menstrual status**								
Premenstrual	280(63.3)	14(3.2)	0.206[0.068,0.617]	0.005	3.417[0.420,27.770]	0.250	0.060[0.007,0.502]	0.009
Postmenstrual	162(36.7)	17(3.8)	2.023[0.263,15.559]	0.499	1.837[0.592,5.696]	0.292	1.101[0.123,9.854]	0.931
**Clinical stage**^a^								
I/II	289(65.4)	14(3.2)	0.881[0.195,3.977]	0.869	5.385[0.695,41.710]	0.107	0.164[0.015,1.805]	0.139
III	153(34.6)	17(3.8)	0.288[0.094,0.884]	0.030	1.240[0.373,4.117]	0.726	0.232[0.062,0.868]	0.030
**Molecular type**								
HR+	280(63.3)	24(5.4)	0.466[0.189,1.149]	0.097	4.439[1.015,19.413]	0.048	0.105[0.022,0.507]	0.005
HER2+	182(41.2)	12(2.7)	0.167[0.044,0.631]	0.008	0.914[0.218,3.828]	0.902	0.182[0.040,0.831]	0.028
TN	83(18.8)	5(1.1)	NA	0.999	0.756[0.126,4.525]	0.759	NA	0.999
** N stage**								
N2/N3	151(34.2)	17(3.8)	0.292[0.095,0.896]	0.031	1.243[0.374,4.127]	0.723	0.235[0.063,0.878]	0.031
N0/N1	291(65.8)	14(3.2)	0.876[0.194,3.953]	0.863	5.374[0.694,41.624]	0.107	0.163[0.015,1.797]	0.139
**T stage**								
T3	29(6.6)	4(0.9)	0.073[0.007,0.819]	0.034	0.204[0.012,3.344]	0.265	0.360[0.032,4.000]	0.405
T2	261(59.0)	17(3.8)	0.729[0.206,2.584]	0.625	2.875[0.644,12.850]	0.167	0.254[0.042,1.518]	0.133
T1	152(34.4)	10(2.3)	0.516[0.099,2.680]	0.431	2.269[0.456,11.289]	0.317	0.227[0.031,1.647]	0.143

*Abbreviations*: *0/I *Grades 0–1, *II/III *Grades 2–3, *IV *Grade 4, *HR *Hazard ratio, *CI *Confidence interval, *HR+ *Hormone receptor-positive, *HER-2+ *Human epidermal growth factor receptor-2-positive, *TN *Three-negative

^a^According to the 7th edition of the UICC/AJCC staging system

NA: Loglik converged before variable 1,2 and coefficient was infinite

**Table 3:** The relationship between the grade of CIL and relapse-free survival (RFS)

Characteristics	Number(n,%)	Events(n,%)	II/III vs. 0/I		II/III vs. IV		IV vs. 0/I	
			HR, 95%CI	*P*-value	HR, 95%CI	*P*-value	HR, 95%CI	*P*-value
**Menstrual status**								
Premenstrual	280(63.3)	38(8.6)	0.821[0.331,2.038]	0.671	1.618[0.697,3.757]	0.263	0.507[0.168,1.535]	0.230
Postmenstrual	162(36.7)	32(7.2)	1.849[0.432,7.922]	0.407	1.237[0.579,2.644]	0.583	1.495[0.327,6.825]	0.604
**Clinical stage**^a^								
I/II	289(65.4)	31(7.0)	2.373[0.546,10.304]	0.249	1.432[0.608,3.376]	0.411	1.657[0.336,8.163]	0.535
III	153(34.6)	39(8.8)	0.673[0.273,1.657]	0.389	1.423[0.677,2.990]	0.352	0.473[0.171,1.304]	0.148
**Molecular type**								
HR+	280(63.3)	45(10.2)	0.936[0.406,2.156]	0.876	1.847[0.872,3.915]	0.109	0.506[0.188,1.368]	0.180
HER2+	182(41.2)	25(5.7)	0.748[0.216,2.592]	0.647	1.484[0.580,3.798]	0.411	0.504[0.124,2.043]	0.338
TN	83(18.8)	13(2.9)	NA	0.998	0.634[0.201,2.007]	0.439	NA	0.998
** N stage**								
N2/N3	151(34.2)	38(8.6)	0.682[0.277,1.679]	0.400	1.592[0.736,3.441]	0.237	0.428[0.152,1.206]	0.108
N0/N1	291(65.8)	32(7.2)	2.348[0.541,10.193]	0.254	1.2511[0.553,2.829]	0.591	1.877[0.390,9.049]	0.432
**T stage**								
T3	29(6.6)	8(1.8)	0.580[0.067,5.046]	0.622	0.435[0.083,2.283]	0.325	1.332[0.120,14.740]	0.815
T2	261(59.0)	44(10.0)	1.145[0.439,2.983]	0.782	1.203[0.598,2.416]	0.604	0.952[0.328,2.760]	0.928
T1	152(34.4)	18(4.1)	1.136[0.238,5.413]	0.873	2.058[0.653,6.482]	0.218	0.552[0.098,3.117]	0.501

*Abbreviations*: *0/I *Grades 0–1, *II/III *Grades 2–3, *IV *Grade 4, *HR *Hazard ratio, *CI *Confidence interval, *HR+ *Hormone receptor-positive, *HER-2+ *Human epidermal growth factor receptor-2-positive, *TN *Three-negative

^a^Acording to the 7th edition of the UICC/AJCC staging system

NA: Loglik converged before variable 1,2 and coefficient was infinite

### Construction of a prognostic model

CIL was shown to be an important independent prognostic factor for OS in univariate and multivariate analyses (Table 4).

**Table 4:** Univariate and multivariate analyses for overall survival (OS)

Characteristics	Univariate Analysis		Multivariate Cox Regression Analysis	
	HR^a^, 95%CI	*P*-value	HR^a^, 95%CI	*P*-value
**Menstrual Status**				
premenopausal				
postmenopausal	2.31[1.14,4.69]	0.021	2.39[1.16,4.91]	0.018
**BMI**				
≤ 25				
> 25	1.04[0.45,2.41]	0.934		
**HR** ^**b**^ **Status**				
Negative				
Positive	1.92[0.83,4.47]	0.128		
**HER2 Status**				
Negative				
Positive	0.90[0.44,1.85]	0.769		
**Ki-67 Index**^a^				
≤ 15%				
> 15%	1.38[0.57,3.38]	0.478		
**T Stage**^b^				
T1				
T2	1.00[[0.46,2.19]	1.000		
T3	2.34[0.73,7.46]	0.152		
** N Stage**^b^				
N0				
N1	4.12[1.15,14.77]	0.030	3.85[1.06,14.01]	0.041
N2	3.86[0.97,15.43]	0.056	3.41[0.82,14.16]	0.091
N3	8.85[2.47,31.74]	< 0.001	6.50[1.72,24.60]	0.006
**LVI**				
No				
Yes	2.64[1.22,5.74]	0.014	1.85[0.81,4.19]	0.143
**CIL**				
0/I				
II/III	0.46[0.19,1.10]	0.082	0.39[0.16,0.95]	0.037
IV	0.23[0.07,0.73]	0.012	0.18[0.06,0.59]	0.004

*Abbreviations*: *HR* ^a ^Hazard ratio, *CI *Confidence interval, *BMI *Body mass index, *HR* ^b ^Hormone receptor, *HER-2 *Human epidermal growth factor receptor-2, *LVI *Lymphatic vessel invaded, *CIL *Chemotherapy-induced leukopenia

^a^Indicating DNA synthetic activity as measured using immunocytochemistry

^b^According to the 7th edition of the UICC/AJCC staging system

**Fig. 2:**
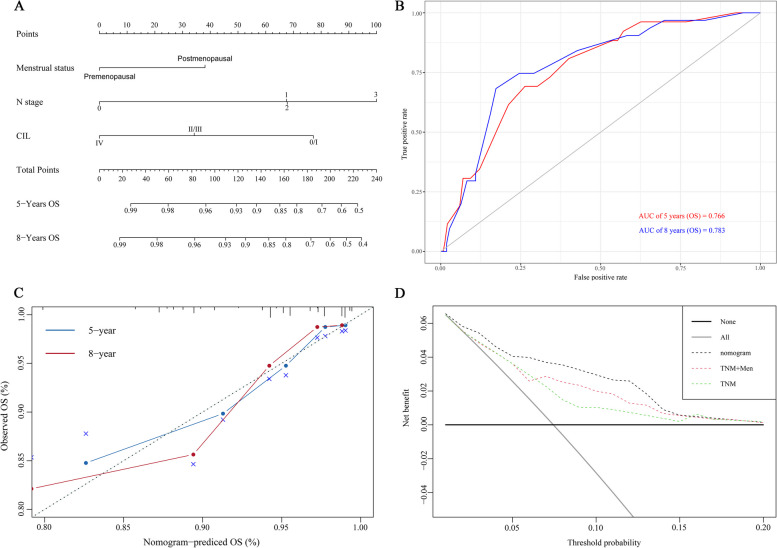
The prognostic model and its validation. **A** A nomogram of the current prognostic model for individualized OS predictions. **B** Receiver operating characteristics (ROC) curves for OS. **C** Calibration plot of the nomogram model at 5- and 8-year for OS. **D** Decision curve analysis (DCA) for the OS.

The variables identified through univariate analysis were inputted into the PH test (Fig. 1C). A prognostic signature for OS was developed using the Cox proportional hazards regression model and stepwise regression analysis (Fig. 1D). Then, all independent prognostic indicators (menstrual status, N stage, CIL grade) were utilized in constructing our model (Fig. 2A).

### Validation of the nomogram

The C-index and ROC curves were used to validate the discrimination, while calibration curves were applied to detect the accuracy of our model. Our model for OS showed a satisfactory predictive accuracy with a C-index of 0.781 (range, 0.705–0.857). Simultaneously, the area under the ROC curve (AUC) achieved satisfactory results for OS (5-year AUC: 0.766; 8-year AUC: 0.783) (Fig. 2B). The calibration curves for 5- and 8-year OS showed good consistency between actual OS and model-predicted OS (Fig. 2C). The DCA curve showed that, compared with the widely used TNM staging system, this prediction model including the CIL grade could provide a better clinical net benefit (Fig. 2D).

### Evaluation of risk stratifications

**Fig. 3:**
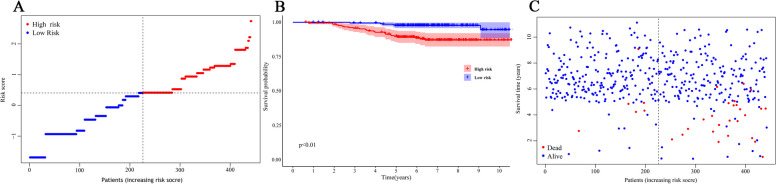
Survival analysis based on risk scores. **A** The distribution and the median value of the risk scores. **B** Kaplan-Meier curves for the OS of patients in the high- and low-risk group. **C** The distributions of OS status, OS and risk scores

The risk score for each patient was calculated using the following formula: Risk score = e^sum (every prognostic factor×corresponding coefficient)^. Then, participants were categorized into low- and high-risk groups based on the median risk score (Fig. 3A). According to the survival analysis, patients in the low-risk group had longer OS compared with those in the high-risk group (Fig. 3B). In the high-risk group, the OS probability at 5 years and 8 years was 88.6% and 87.1%, respectively. Whereas the corresponding probability in the low-risk group was 97.7% and 94.6%, respectively. Consistently, the high-risk group carried a higher risk of death (Fig. 3C).

## Discussion

A total of 442 patients with EBC given regimens of adjuvant chemotherapy containing anthracycline and taxane were enrolled in our study. Patients with grade 2–4 (moderate and severe) CIL had significantly longer OS than those with grade 0–1 (mild) CIL. Patients with severe CIL (grade 4) had significantly longer OS than those with mild CIL. Patients who suffered moderate CIL (grade 2–3) tended to have longer OS than those with mild CIL. However, we did not find a significant difference in OS between patients with moderate CIL and those with severe CIL. Among patients with premenopausal, HER2+, N2/N3 stage or T3 stage, severe or moderate CIL were associated with longer OS compared with those with mild CIL. Similar to results for the entire cohort, there was no significant difference in OS between severe- or moderate-CIL groups.

Consistent with the results published by Han and colleagues [27], moderate or severe CIL were associated with longer survival than mild CIL. Nevertheless, severe CIL would increase the risk of infection and subsequently, the risk of death, compared to moderate CIL [28]. Hence, it may be possible to reduce the dose of follow-up treatment in patients with severe CIL to achieve moderate levels, potentially reducing the risk of infection and improving the efficacy of the anti-tumor ability of other blood cells. Similarly, for patients with mild CIL, increasing the chemotherapy dose to attain moderate CIL may improve the chances of survival. Collectively, in terms of survival and safety, moderate CIL appeared to the best “choice”, especially for patients with high risk factors (e.g., stage III (T3 or N2), premenopausal, HER2+). To confirm our hypothesis, we plan to conduct a study to compare the impact of these two modes on patient survival: administration based on BSA vs. real-time dose adjustment to achieve moderate CIL in patients with EBC.

The relationship between CIL grade and survival could be explained by the pharmacokinetics (PK) and pharmacodynamics (PD) of chemotherapy [29–31]. Cui et al. proposed that the PK of anthracycline may be individualized and associated with single-nucleotide polymorphisms (SNPs) [32]. Several studies have reported that the metabolism of paclitaxel and anthracyclines is catalyzed by cytochrome P450 (CYP450) enzymes, which were encoded by multiple families of CYP genes [33–37]. Therefore, we postulate that SNPs and polymorphisms and mutations in CYP450 genes among different individuals would lead to changes in drug metabolism and, ultimately, to differences in efficacy and AE prevalence. Also, the liver function, kidney function, age, and drug interactions of individual patients may have important roles in these pathways. This hypothesis also shows that the method of administering drugs based solely on BSA is not entirely suitable, and that in some patients, more personalized administration may be required to obtain a better curative effect.

In addition, distinct tumor microenvironments (TME) may exist in patients with varying levels of CIL. The TME is composed of immune cells, stromal cells including cancer-associated fibroblasts, pericytes and mesenchymal stromal cells, extracellular matrix (ECM), growth factors, cytokines, chemokines, and extracellular vesicles, blood vessels and lymphatics. These components interact with each other and the cancer cells. Immune cells play a crucial role in the tumor microenvironment and are divided into lymphoid-derived and myeloid-derived immune cells based on their origin. Numerous myeloid-derived immune cells influence tumor proliferation, survival, differentiation, dissemination, invasion, angiogenesis, TME remodeling, immune regulation, and response to cancer treatment. The major myeloid-associated cells include myeloid-derived suppressor cells (MDSCs), monocytes, tumor-associated macrophages (TAMs), dendritic cells (DCs), and tumor-associated neutrophils (TANs). MDSCs—a heterogeneous group of immature myeloid cells—are associated with breast cancer stage, metastatic tumor size, and chemotherapy efficacy [38]. MDSC levels are closely related to the stage of breast cancer, the size of metastatic tumors, and the effectiveness of chemotherapy. Research shows that MDSC can increase the risk of breast cancer recurrence and metastasis after surgery, and patients with low levels of MDSC have better chemotherapy outcomes [39, 40]. Furthermore, MDSCs can induce IL-1β and IL-17 production, signaling between tumor cells and macrophages leading to tumor cell growth and invasion potentiation. TAMs secrete CCL22 attracting Tregs while also enhancing their function through TGF-β secretion. Human TAMs boost the progression of EGF tumors [41, 42]. TAMs upregulate matrix metalloproteinases (MMPs), disrupting interstitial collagen, increasing collagen synthesis and assembly, leading to TME reconfiguration that favors tumor spread [43]. Numerous studies have reported that TAN promotes tumor cell expansion, migration, and invasion. TAN can release particulate components such as elastase to promote cancer cell proliferation/invasion. Additionally, TAN-secreted IL-1β and MPs promote tumor cell exodus towards premetastatic niches for invasion and metastasis [44, 45]. Several studies have shown that when chemotherapy drugs kill tumor cells in micro metastases, they also damage immune cells and affect the tumor immune microenvironment [46, 47]. We speculate that in patients with severe CIL, tumor-associated myeloid cells (MDSC, TAM, TAN, etc.) may be suppressed to a greater extent, thereby weakening their ability to promote metastasis and colonization growth of small tumor foci, improving the prognosis for some patients. On the contrary, in patients who do not suffer or suffer mild CIL, the degree of suppression of tumor-associated myeloid cells may be even lower, thereby reducing their ability to promote metastasis and colonization growth of small tumor foci, resulting in a poor prognosis for these patients.

Univariate and multivariate analyses showed that menstrual status and N stage were independent predictors for OS. With respect to menstrual status, Bezword and colleagues showed that estrogen could promote the transition of BC cells from the G0/G1 stage to the S-G2/M of the cell cycle to increase the sensitivity to chemotherapy [48]. Blockade of estrogen action can inhibit BC-cell proliferation and antagonize the cytotoxic effect of chemotherapy drugs. With regard to the N stage, tumor cells invade adjacent tissues locally initially and then enter the microvascular system composed of lymphatic and blood systems. Then, tumor cells can transfer to distant tissues and organs through the lymphatic circulation [49]. Hence, the greater the invasion of lymph nodes, the greater is the probability and risk of distant metastasis.

Pashtoon and colleagues revealed that chemotherapy-induced neutropenia (CIN) might be a prognostic factor among patients with solid tumors (including BC) [50]. Furthermore, Han et al. found that CIN was closely associated with OS. They discovered CIN to be an independent prognostic indicator among 335 patients with EBC receiving six cycles of cyclophosphamide, epirubicin, and fluorouracil (CEF) [27]. However, our study had three main advantages compared with their study. First, the therapeutic regimen of A and T is more prevalent than the CEF regimen for current treatment options of EBC [51]. Therefore, our study probably has relatively higher clinical relevance. Second, the number of patients with EBC in our study was greater than that in their study, which increases the strength of the statistical analyses of our study. Third, in our study, a nomogram was established to visualize the data related to survival probability, thereby making it easier to guide clinical practice.

However, our study had three main limitations. First, our study was retrospective, and sample-selection bias might have been present. Second, all patients with EBC were enrolled from one center (SYSUCC), which hinders the generalizability of our results. Third, we assumed that SNPs, NETs, as well as polymorphisms and mutations in CYP450 genes were probably involved in cancer relapse and metastasis, but we failed to measure levels of these markers. In the future, expression of CYP450 genes and numbers of NETs should be measured in patients suffering from EBC.

## Conclusions

The grade of CIL was strongly associated with the prognosis among patients with EBC who received a regimen containing both anthracyclines and taxanes. Patients with a “moderate” CIL grade tended to have better survival outcomes.

### Supplementary Information

**Additional file 1: Supplement Appendix 1.** The inclusive criteria and excluded criteria.

## Data Availability

The data analyzed in this study are available from the corresponding author (Zhong-Yu Yuan, E-mail: yuanzhy@sysucc.org.cn) on reasonable request.

## Abbreviations

CIL: Chemotherapy-induced leukopenia
EBC: Early breast cancer
A: Anthracycline
T: Taxanes
OS: Overall survival
HR: Hazard ratio
CI: Confidence interval
HER2+: Human epidermal growth factor receptor 2-positive
BC: Breast cancer
MT: Molecular type
BSA: Body surface area
AEs: Adverse effects
WBCs: White blood cells
G-CSF: Granulocyte colony-stimulating factor
SYSUCC: Sun-Yat Sen University Cancer Center
ER: Estrogen receptor
PR: Progesterone receptor
HER2: Human epidermal growth factor receptor 2
PG: Pathological grade
IHC: Immunohistochemistry
ERBB2: Erb-B2 receptor tyrosine kinase 2
FISH: Fluorescence in situ hybridization
RFS: Relapse free survival
PH: Proportional Hazards
C-index: Concordance index
ROC: Receiver operating characteristic
DCA: Decision curve analysis
BMI: Body mass index
TNM: Tumor–node–metastasis
QOL: Quality of life
PK: Pharmacokinetics
PD: Pharmacodynamics
SNPs: Single Nucleotide Polymorphisms
CYP450: Cytochrome P450
TME: Tumor microenvironments
ECM: Extracellular matrix
MDSCs: Myeloid-derived suppressor cells
TAMs: Tumor-associated macrophages
DCs: Dendritic cells
TANs: Tumor-associated neutrophils
MMPs: Matrix metalloproteinases
CIN: Chemotherapy-induced neutropenia
CEF: Cyclophosphamide, epirubicin, and fluorouracil
