# Supplementary material for: “Moderate” adjuvant chemotherapy-induced leukopenia is beneficial for survival of patients with early breast cancer: a retrospective study
Source: BMC Cancer. 2023 Dec 13;23:1227. doi: 10.1186/s12885-023-11680-x (PMC10720186; doi:10.1186/s12885-023-11680-x)
Supplement: Supplementary file 1 — Additional file 1: Supplement Appendix 1. The inclusive criteria and excluded criteria. [file 12885_2023_11680_MOESM1_ESM.docx]

**Supplement appendix1.** The inclusive criteria and excluded criteria.

The inclusive criteria:

1) age ≥ 18 years old;

2) pathological diagnosis as invasive BC;

3) without distant metastasis (lung, bone, liver, and brain);

4) patients receiving all cycles of chemotherapy in our hospital;

5) the chemotherapy regimens containing anthracyclines and taxanes;

6) available complete baseline laboratory data and specific follow-up data.

The excluded criteria:

1) were pregnant or breastfeeding:

2) had a pathological diagnosis of ductal carcinoma in situ;

3) had synchronal malignancies;

4) the dose of drug used below 85% of standard dose;

5) had taken any medicine inducing an immune or inflammatory response in the most recent 3 months;

6) had any inflammatory disease (autoimmune diseases included).
